# Use of artificial intelligence in healthcare in South Africa: A scoping review

**DOI:** 10.4102/hsag.v30i0.2977

**Published:** 2025-07-14

**Authors:** Jennifer Chipps, Thandazile Sibindi, Amanda Cromhout, Antoine Bagula

**Affiliations:** 1 School of Nursing, Faculty of Community Health Sciences, University of the Western Cape, Cape Town, South Africa; 2 Department of Computer Science, Faculty of Natural Sciences, University of the Western Cape, Cape Town, South Africa

**Keywords:** artificial intelligence, deep learning, health sector, machine learning, South Africa

## Abstract

**Background:**

Artificial intelligence (AI) transformed healthcare worldwide and has the potential to address challenges faced in the South African healthcare sector, such as limited public institutional capacity, staff shortages, and variability in skills levels that exacerbate the demand on the healthcare system that can lead to compromised care and patient safety.

**Aim:**

This study aimed to describe how AI, especially machine learning is used in healthcare in South Africa over the last 5 years.

**Method:**

The Joanna Briggs Institute (JBI) methodology for scoping reviews was used. Peer-reviewed articles in English, which were published from 2020 to date were sourced and reviewed using the Population, Concept, Context (PCC) framework.

**Results:**

A total of 35 articles were selected. The results showed a focus on conventional machine learning, a health focus on HIV and/or tuberculosis (TB) and cancer, and a lack of big data in fields other than cancer.

**Conclusion:**

There has been an increase in the use of machine learning in the analysis of health data, but access to big data appears to be a challenge.

**Contribution:**

There is a need to have access to high-quality big data, inclusive policies that promote access to the benefits of using machine learning in healthcare, and AI literacy in the health sector to understand and address ethical implications.

## Introduction

South Africa has a quadruple burden of diseases, which severely impacts the delivery of healthcare (De Villiers 2021; Louw et al. 2023; Okeibunor et al. 2023). Healthcare is often inaccessible to people living in remote areas (De Villiers 2021; Louw et al. 2023), and most people depend on limited public resources. Limited public institutional capacity, staff shortages, high staff turnover rates, and variability in skills levels exacerbate the demand on the healthcare system and can lead to compromised care and patient safety (De Villiers 2021).

Artificial intelligence (AI) has been posited to address some of these challenges. Artificial intelligence refers to the imitation of human intelligence by automated processes (Kuziemsky et al. 2019) such as the use of machine learning (i.e., training machines to learn from datasets and performs tasks; Shandhi & Dunn 2022) and the use of generative AI (i.e., the use of large language models to do a range of tasks). Artificial intelligence has been applied in healthcare to assist in the diagnoses of diseases, analyse healthcare plans, monitor health, develop personalised treatment plans, and perform surgical treatment through robotics (Amisha et al. 2019). Artificial intelligence shows potential for personalised medicine as individual risk can be predicted from patient data that enables the development of individualised treatment plans, which can enhance patient outcomes (Amisha et al. 2019; Kuziemsky et al. 2019; Shandhi & Dunn 2022). Artificial intelligence can also streamline healthcare delivery and provide more efficient healthcare, for example, online appointment scheduling, online check-ins, digitalisation of medical records, and automatic reminders for follow-up appointments (Amisha et al. 2019).

Within the field of AI, machine learning (ML) has been used in predictive analytics where predictive models are formed by combining machine learning and traditional statistics and are used for prognosis, optimising healthcare delivery, and individualised treatment (Manlhiot 2018). Machine learning includes conventional machine learning (which focuses on developing algorithms and models that permit computers to learn from information); deep learning (which uses neural networks to simulate the human brain to learn from unstructured data, such as images and speech recognition); and ensemble learning (which combines multiple models to improve accuracy and robustness), and can be applied to both conventional and deep learning models (Sharifani & Amini 2023). Machine learning models are useful to determine which patients are likely to benefit from a particular treatment based on certain characteristics (e.g., genetically informed therapeutic planning) (Dong et al. 2015; Shandhi & Dunn 2022) and to identify biomarkers that can assist in early detection of disease, prediction of treatment response, and provide indicators of the progression of diseases (Shandhi & Dunn 2022).

### Review question

The following is the review question: ‘*What articles on AI used in healthcare in South Africa were published in the last 5 years?*’ The objectives were to determine: (1) what the status of using AI in the South African health system is, and (2) what applications of AI-based innovations are currently in use in the health sector in South Africa. As no generative AI articles were found, the review focused on machine learning.

## Materials and methods

### Research design

This scoping review followed the guidelines of the Joanna Briggs Institute (JBI) for evidence-synthesis (Aromataris et al. 2024; Peters et al. 2020), and the reporting is done in accordance with the Preferred Reporting Items for Systematic Reviews and Meta-Analyses extension for scoping reviews (PRISMA-ScR) checklist (Tricco et al. 2018).

### Protocol and registration

The review protocol was registered in Open Science Framework (OSF) prior to conducting the study, and is available at https://doi.org/10.17605/OSF.IO/2SMU4

### Eligibility criteria

Given the rate of development of technology, this scoping review collected and analysed peer-reviewed full-text empirical research articles written in English from 2020 to 2024 to ensure that only the latest research articles pertaining to the use of AI in healthcare settings in South Africa are included. All study methods were considered, except non-research studies such as reviews and opinion article. Using the PCC (population, concept, context) framework (Peters et al. 2020), inclusion and exclusion criteria were formulated (Table 1). The common types of machine learning are defined in Appendix 2. Studies that did not comply with the inclusion criteria were excluded.

**TABLE 1:** Inclusion and exclusion criteria based on participants, context, and concept.

Terms	Inclusion	Exclusion
Participants	Studies where all patients or records are in South African health care setting	South African patients identified as part of an international multi-country study
Context	South African health service (e.g., hospitals, clinics, communities, or research settings, medical institutions)	-
Concept	Conventional machine learning, deep learning and ensemble learning	Standard statistical analysis, generative AI

AI, artificial intelligence.

### Search strategy, selection of sources, and data extraction

We used the key terms of AI, machine learning and deep learning synonyms, South Africa and health sector (see Appendix 1, Table 1-A1 for detailed search strategy), to conduct searches on PubMed, Academic search Complete, and Google Scholar, to retrieve the most relevant data. Descriptors such as titles, abstracts, and full texts of relevant and suitable articles on the topic were used to conduct the search. Relevant studies from the reference lists of retrieved articles were hand-searched from Google Scholar database (‘snowballing’).

Following the literature search, all identified sources were imported to the systematic review management software, namely Covidence (2023), which guided the screening process comprising the screening of titles and abstracts, review of full texts, and data extraction. Two reviewers (the third and fourth authors) screened the sources independently for eligibility. Disagreements between the reviewers at each stage of the selection process were resolved through online discussion by the reviewers and checked by the first author. The PRISMA-ScR flowchart was used to illustrate the search process (the removal of duplicates, the number of qualifying sources included for analysis, and the reason for excluding non-eligible sources). Using a data extraction tool in Excel, data were extracted by the fourth author, and checked by the first author. There was no formal quality assessment of the sources included in the review because a formal assessment of the methodological quality is usually not required for scoping reviews (Peters et al. 2020).

### Data analysis

For scoping reviews, frequency counts for the required fields of data are sufficient, although a more in-depth analysis can be appropriate (Peters et al. 2020). Frequency counts and thematic analysis Braun and Clarke (2006) were performed by the first author. Themes of health area, source of data and type of machine learning were analysed.

### Ethical considerations

This article followed all ethical standards for research without direct contact with human or animal subjects.

## Review findings

### Selection of sources of evidence

**FIGURE 1:**
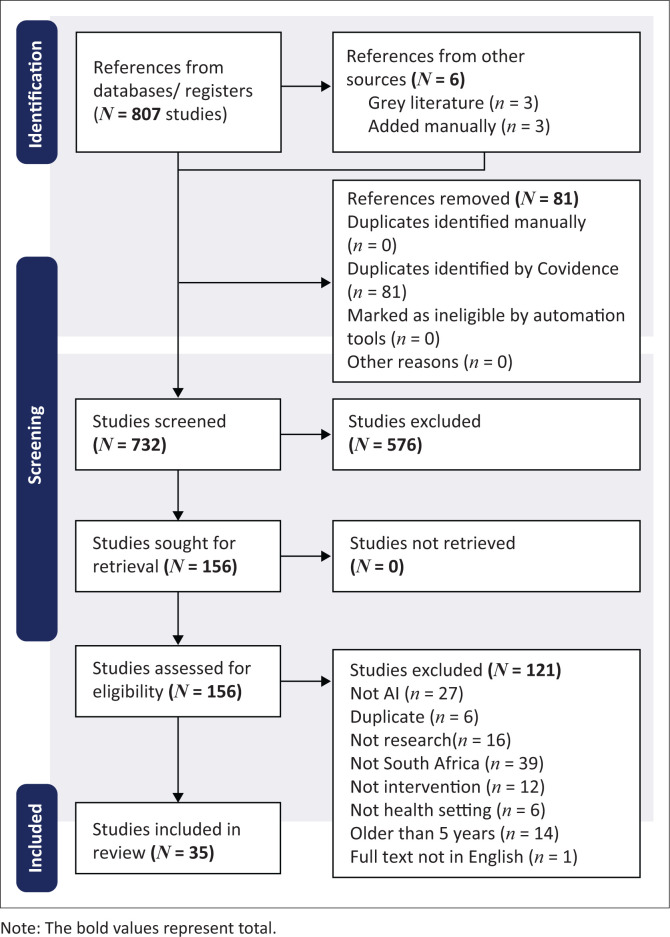
PRISMA-ScR flowchart illustrating the search process.

A total of 810 records were retrieved for the period from 2020 to 2024, with one current unpublished study included. Thirty-five articles (*n* = 35) were selected for final analysis (Figure 1). Of the 35 articles, 9 (25.7%) were published between 2020 and 2021, and 26 (74.3%) between 2022 and 2025 (Table 2).

**TABLE 2:** A summary of articles (*N* = 35).

Variables	Frequency	
	*n*	%
**Year**		
2020–2021	9	25.7
2022–2024	26	74.3
**Health area**		
HIV or TB	8	24.2
Cancer	7	21.2
COVID-19	7	21.2
Other	11	33.3
**Data**		
Primary data	14	42.4
Registry	11	33.3
Other	8	24.2

COVID-19, coronavirus disease 2019; HIV, human immunodeficiency virus; TB, tuberculosis.

#### A summary of the studies included in the review

Three articles were on the same study focusing on developing a machine learning algorithm to predict mortality in critically ill children (Pienaar et al. 2022a, 2022b, 2023). Most data were primary data (*n* = 14, 42.4%) or from established data registries such as the South African Cancer Registry, the National Health Laboratory Services (NHLS) and the World Data registry of COVID-19 cases (Table 2).

### Characteristics of the studies

The reviewed studies used a range of conventional machine learning (50%), deep learning (20.2%) and ensemble learning techniques (29.8%) focusing on different health areas (Table 3). Two health areas had the highest number of studies: cancer and COVID-19 (*n* = 7, 21.2% each) (Table 2). All the studies on cancer used data from the South African Cancer Registry. Separate studies were conducted by groups with overlapping authors who work in the field, with three studies by Achilonu et al. (2021a, 2021b, 2022) and two studies by Olago et al. (2020, 2023) (Table 3). The purpose of using conventional and ensemble machine learning ranged from classifying risk for length of stay (Achilonu et al. 2021a), generating histopathology reports (Olago et al. 2020) and cancer diagnoses related to human immunodeficiency virus (HIV) diagnoses (Olago et al. 2023) and processing free text to classifying benign versus malignant cancer (Achilonu et al. 2021b, 2022). Deep learning was used to generate malignant urine cytology images (McAlpine et al. 2022), and the prediction of the Gleason Grade group in prostate cancer (Mokoatle et al. 2022).

**TABLE 3:** Characteristics of the studies.

Author	Year	Purpose	Area of health	Type of data	Source of data	ML method	ML type
Achilonu et al.	2021	To develop logistic regression (LR) and support vector machine (SVM) models that differentiate low from high risk for prolonged hospital length of stay (LOS) following colorectal cancer resection	Cancer	Registry	South African Cancer Registry (CRCSA), Johannesburg, South Africa	SVM, ANN, RF, C5.0, NB, GLM	Conventional and deep ML
Achilonu et al.	2022	To develop a rule-based natural languageprocessing algorithm from free text for female breast cancer subtypes	Cancer	Registry	Breast cancer pathology reports in South African NHLS	GPR	Conventional ML
Achilonu et al.	2021	To assess the effectiveness of randomforest (RF) and support vector machine (SVM) in the classification of reports into benign and malignant classes from colorectal, breast and prostate cancer free text reports	Cancer	Registry	South African National Cancer Registry, National Health Laboratory Service (NHLS)	RF, SVM	Conventional ML
McAlpine et al.	2022	To develop and assess a generative adversarial network (GAN) model for generating realistic, morphologically diverse malignant urine cytology images to enhance limited medical imaging datasets	Cancer	Registry	26 malignant urine cytology slides, derived from two different cytology laboratories:National Health Laboratory Service (NHLS), Lancet Laboratories	GAN	Deep ML
Mokoatle et al.	2022	Application of machine learning and biological experiments to validate the performance of the deep learning system associated with the prediction of the Gleason grade group in prostate cancer	Cancer	Registry	Southern African Prostate Cancer Study (SAPCS)	XGBOOST, LSTM, RF, RNN	Conventional, ensemble and deep ML
Olago et al.	2020	To evaluate how each machine learning model performs in the task of classifying histopathology reports	Cancer	Registry	National Cancer Registry, National Health Laboratory Service (NHLS)	KNN, RF, SVM, GLM, AdaBOOST, NB, SGD, DT	Conventional and ensemble ML
Olago et al.	2023	To map the place of cancer diagnosis in relation to HIV care centre among people living with HIV within South Africa	Cancer	Registry	National Health Laboratory Service (NHLS) that linked to cancer record at the National Cancer Registry (NCR)	NB, AdaBOOST, GLM, SGD, KNN, C5	Conventional and ensemble ML
Mpanya et al.	2022	To demonstrate the utility of machine learning in predicting all-cause mortality in heart failure patients hospitalised in a tertiary academic centre	Cardiovascular	Database	PMR Cardio database, Charlotte Maxeke Johannesburg Academic Hospital, South Africa	RF, SVM, GLM, XGBOOST, ANN (MLP), DT	Conventional, ensemble and deep ML
Chimbunde et al.	2023	To quantify COVID-19 risk factors and predict COVID-19 intensive care unit (ICU) mortality in South Africa based on machine learning algorithms.	COVID-19	Hospital records	Data collected from SARS-CoV-2-infected patients treated at Tygerberg Hospital, Cape Town	ANN, RF	Ensemble and deep ML
Fuller et al.	2023	To develop a model to predict adverse outcome in patients presenting with suspected COVID-19	COVID-19	EHR	ED HECTIS, routine data emergency department, Western Cape, South Africa	XGBoost	Ensemble ML
Lieberman et al.	2023	To distinguish between hot-spots and areas characterised by stochastic spreading of COVID-19 cases	COVID-19	Registry	Daily data provided by Gauteng Department of Health to National Institute of Communicable Diseases (NICD)	GMM	Conventional ML
Okonkwo et al.	2022	To introduce COVID-Bot to help screen students and confirm their COVID-19 vaccination status	COVID-19	Primary data	Primary data obtained from enrolled students (Johannesburg, South Africa)	NLP	Conventional ML
Stevenson et al.	2021	To develop a functional alert system for an additional wave of COVID-19 cases	COVID-19	Registry	Real-world data from between the first and second waves of COVID-19 from NHLS	RNN, LSTM	Deep ML
Akinola et al.	2023	Time-series forecasting of COVID-19 cases	COVID-19	Registry	Our World in Data, a publicly available repository on COVID-19 daily case counts in South Africa	RNN	Deep ML
Kolozali et al.	2024	To explore the potential of Internet of Things (IoT) devices and explainable artificial intelligence (AI) techniques in predicting biomarker values associated with gestational diabetes mellitus (GDM) when measured 13 to 16 weeks prior to diagnosis.	Diabetes	Primary data	Primary data were collected from 17 mothers at their enrolment visit to Chris Hani Hospital, Johannesburg, South Africa	RF, DT, CMTF Elastic Net	Conventional and ensemble ML
Eken et al.	2020	To evaluate the feasibility of a novel markerless movement tracking system based on a machine-learning algorithm as an alternative for human gait analyses	HIV/TB	Primary data	Cross-sectional cohort (*n* = 5) of girls with HIV encephalopathy and five typically developing girls (Cape Town, South Africa)	CNN	Deep ML
Esra et al.	2023	Validation and improvement of a machine learning model to predict interruptions in antiretroviral treatment in South Africa	HIV/TB	Hospital records	Data from patients receiving ART at facilities in the Gauteng and North West provinces, South Africa	AdaBoost, GLM	Conventional and ensemble ML
Esra et al.	2023	Historical visit attendance as predictor of treatment interruption in South African HIV patients: Extension of a validated machine learning model	HIV/TB	Hospital records	Data from patients receiving ART at facilities in the Gauteng and North West provinces, South Africa	CatBoost	Ensemble ML
Majam et al.	2023	To evaluate the accuracy of a ML-based risk assessment tool, trained using data collected from a digital survey, in assessing HIV risk in those believed to be negative or unaware of their HIV status	HIV/TB	Primary data	Primary data from a cross-sectional and cohort studies from Gauteng, Mpumalanga, KwaZulu-Natal, South Africa	GLM, SVM, GBDT, DT	Conventional and Ensemble ML
Maskew et al.	2022	To determine whether machine learning applied to routinely collected longitudinal HIV phenotypic and clinical outcome data in South African programmes could consistently identify patients at risk of poor outcomes in terms of two key programmatic outcomes: (1) attendance at next scheduled clinic visit and (2) suppression of next HIV viral load (VL).	HIV/TB	Primary data	Longitudinal data from patients accessing HIV care and treatment at public sector treatment sites in Mpumalanga and the Free State	GLM, AdaBOOST, RF	Conventional and ensemble ML
Onywera et al.	2020	To classify the penile microbiota of HPV-infected men and the impact of HIV status	HIV/TB	Primary data	Cross-sectional data from 2-year longitudinal HPV couples cohort study, Gugulethu, South Africa	LEfSe	Conventional ML
Pahar et al.	2021	To train and evaluate five machine learning classifiers to distinguish between the coughs of TB patients and the coughs of patients suffering from other lung ailments and for whom TB was excluded as a diagnosis	HIV/TB	Primary data	Primary data collected from patients at a TB clinic, Cape Town, South Africa	CNN, SVM, KNN, ANN, GLM	Conventional and deep ML
Sibandze et al.	2020	To use diagnostic ML algorithms to determine if there are clear patterns among the different anatomic sites impacting drug resistance and/or genotypic clustering of Mtb isolates in affected individuals.	HIV/TB	Registry	A total of 75 culture-positive isolates confirmed by National Health Laboratory Services (NHLS)	CART, RF, MARS, XGBOOST, SGD	Conventional and ensemble ML
Turbe et al.	2021	To determine the use of deep learning to classify images of rapid HIV tests as positive or negative	HIV/TB	Primary data	Primary data collected of 11,374 photographs of HIVRDT, KwaZulu-Natal, South Africa	CNN	Deep ML
Martineau et al.	2022	To identify and make use of large-scale climatic precursors of malaria outbreaks using an extensive, but cost-effective suite of machine-learning techniques designed to provide early malaria warnings with lead times up to two to three seasons with high accuracies	Malaria	Database	Observational data set from Malaria Institute, Tzaneen, South Africa	GMM, GLM, SVM, GBDT, RF, NB and XGBOOST, AdaBoost	Conventional and ensemble ML
Sandstrom et al.	2022	To investigate the performance of a machine learning approach (CNN) for otitis media screening using digital otoscopic images of tympanic membranes diagnosed by an expert panel	Otitis media	Primary data	Digital images of tympanic membrane collected in a prospective study in Tshwane (Pretoria), Gauteng, South Africa at one district hospital ENT clinic, three primary healthcare clinics and one itinerant clinic for school screening	CNN	Deep ML
Pienaar et al.	2022	Comparison of artificial neural network models developed to predict a composite outcome of death before discharge from hospital or admission to the PICU	Paediatric critical illness	Primary data	Primary data collected from patients seen at the tertiary hospital, Free state, South Africa	GLM, ANN, XGBOOST	Ensemble and deep ML
Pienaar et al.	2022	Development of an artificial neural network (ANN) model for the prediction of mortality in two tertiary paediatric intensive care units	Paediatric critical illness	Primary data	Primary data from paediatric patients admitted during the study period, Free State	ANN, GLM,	Conventional and ensemble ML
Pienaar et al.	2023	To describe how domain knowledge was elicited, including the use of a documented literature search and Delphi procedure for a ML model for paediatric illness	Paediatric critical illness	Primary data	Primary data collected from three paediatric intensivists, six specialist paediatricians and three specialist anaesthesiologists from a single centre tertiary hospital, Free State, South Africa	None	None
Nel et al.	2022	To develop an automated screening tool to predict SAM risk from WFA growth curves, and to determine its predictive ability compared with simple changes in weight or WFA score	Severe acute malnutrition	Primary data	Training data developed from survey of child growth experts, compiling growth curves to diagnose severe acute malnutrition (SAM) Validated against SAM (cases) recruited from two public-sector hospitals in Tshwane district, Gauteng province, South Africa	ANN	Deep ML
Tokac et al.	2025a; 2025b	Predicting readmissions in surgical and trauma patients at an emergency department in KwaZulu-Natal	Surgery and trauma	EHR	The HEMR database of surgical and trauma admissions at Greys Hospital, KwaZulu-Natal	RF, NLP	Conventional and ensemble ML
Van Zyl-Cillié et al.	2024	To develop a supervised machine learning model to identify the factors that are most strongly associated with burnout and emotional exhaustion in nurses.	Mental health	Primary data	Training data developed from cross-sectional survey data collected from all categories of nursing staff working in public and private hospitals. Only medical-surgical data was used.	NB ETC, Ligh GBM, RF, Ada Boost	Conventional ML, ensemble ML
Smith et al.	2021	To identify patient factors, physiological and time-related factors, which place patients into a group at increased risk of mortality	Surgery and trauma	EHR	The HEMR database of surgical and trauma admissions at Greys Hospital, KwaZulu-Natal	DT	Conventional ML
Pienaar et al.	2024	The development of predictive models for triage or identification of critically ill children presenting to a tertiary hospital.	Trauma and paediatric critical illness	Primary data	Primary data collected prospectively for 6 months for children < 13 presenting at ED with acute illnesses	3xANN; 3 XGBs & 3 LR	Conventional and ensemble ML, and deep ML

Note: Please see the full reference list of this article, https://doi.org/10.4102/hsag.v30i0.2977 for more information.

AdaBoost, adaptive boosting; ANN, artificial neural networks; CatBoost, categorical boosting; CNN, convolutional neural networks; CART, classification and regression trees; CMTF, coupled-matrix tensor factorisation; C5.0, decision tree algorithm; DT, decision tree; ETC, extra trees classifier; XBoost, extreme gradient boosting; GBC, gradient boosting classifier; GPR, Gaussian process regression; GAN, generative adversarial networks; GBDT, gradient boosted decision trees; GLM, generalised linear model; GMM, Gaussian mixture model; ML, machine learning; KNN, K-nearest neighbour; LEfSe, linear discriminant analysis effect size; Light GBM, light gradient boosting machine; LSTM, long short term memory; MARS, multivariate adaptive regression splines; NB, naive bayes; RNN, recurrent neural networks; NLP, natural language processing; SGD, stochastic gradient descent; SVM, support vector machine; RF, random forest.

Studies on COVID-19 were also common, because of the recent pandemic (Table 3). Data sets were obtained from the National Institute of Communicable Diseases (NICD) (Lieberman et al. 2023), the National Health Laboratory Service (NHLS) (Stevenson et al. 2021) and Our World in Data (Akinola et al. 2023), a publicly available repository on COVID-19 daily case counts in South Africa. The rest of the studies used either primary data (Okonkwo, Amusa & Twinomurinzi 2022), hospital records (Chimbunde et al. 2023) or data from the Hospital and Emergency Centre Tracking Information System application (HECTIS7), which is the emergency department electronic register in the Western Cape (Fuller et al. 2023). Deep learning and machine learning were used to predict ICU mortality (Chimbunde et al. 2023) and forecast new waves of COVID-19 (Akinola et al. 2023; Stevenson et al. 2021), and conventional and ensemble machine learning were used to confirm vaccination status (Okonkwo et al. 2022), classify hot spots (Lieberman et al. 2023) and predict adverse outcomes (Fuller et al. 2023).

The next most common health area targeted with machine learning was HIV and/or tuberculosis (TB) (*n* = 8, 24.2%) (Table 2). Six of the eight studies used primary data collected from observational studies (Eken et al. 2020; Majam et al. 2023; Maskew et al. 2022; Onywera et al. 2020; Pahar et al. 2021; Turbé et al. 2021), two studies used hospital records of patients receiving anti-retroviral therapy (ART) treatment (Esra et al. 2023a, 2023b), and one study used data on culture positive isolates confirmed by the National Health Laboratory Services (NHLS) (Achilonu et al. 2021a) (Table 3). The purpose of using machine learning ranged from using deep learning to develop movement tracking systems (Eken et al. 2020), classifications of TB-related versus other coughs, assessing the impact of HIV on Human papillomavirus (HPV) (Onywera et al. 2020) to the classification of rapid HIV tests (Turbé et al. 2021). Conventional and ensemble machine learning were used to classify and predict poor outcomes from HIV such as interruptions to ART treatment (Esra et al. 2023a, 2023b), attendance at clinic visit and viral load (Maskew et al. 2022), HIV risk (Majam et al. 2023), and drug resistance (Sibandze et al. 2020).

The rest of the studies (*n* = 9, 27.2%) focused on a range of different health focus areas (Table 3). Three studies conducted by the same group of authors, specifically focused on paediatric critical illnesses, using primary data and deep learning to develop a model to predict mortality in paediatric ICUs (Pienaar et al. 2022a), followed by a comparison of different models using conventional and ensemble machine learning (Pienaar et al. 2022b). Thereafter, a study was performed by experts to describe the domain knowledge for a machine learning model for paediatric illness (Pienaar et al. 2023). Deep learning was also used to screen digital otoscopic images (44) and a screening tool for severe acute malnutrition (Nel et al. 2022) (Table 3). Conventional and ensemble machine learning were further used to predict mortality in heart failure (Mpanya et al. 2023), risk of readmission in surgical and trauma emergency departments (Tokac et al. 2025a, 2025b), and to identify risk factors for mortality in laparotomy surgery (Smith et al. 2022), gestational diabetes (Kolozali et al. 2024) and provide malaria warnings (Martineau et al. 2022). In one paper, a combination of deep learning, ensemble and conventional learning was used to develop the most effective model for triage of critically ill children presenting to a tertiary hospital (Pienaar et al., 2022b).

Lastly, a range of AI methods used in the reviewed studies fall into three main types based on their characteristics and applications (Table 1-A2). Conventional machine learning was most often used (22, 66.7%) among the articles, usually combined with ensemble learning (12/22) or deep learning (2/22) or in a combination of all three (4/22) (Table 3). Conventional machine learning relies on traditional statistical and mathematical approaches and is used for tasks such as classification and regression, often in simpler datasets and problems where feature extraction is typically performed manually. Ensemble boosting was used in 18 (54.5%) articles, often in combination with deep or conventional machine learning (14/18) (Table 3). Ensemble learning combines multiple models, such as decision trees, to enhance prediction accuracy, robustness, and generalisability, which are useful in managing diverse and imbalanced datasets. Lastly, deep learning, which is characterised by its reliance on neural networks with multiple layers, designed to process large and complex datasets, was used in 14 articles (42.4), eight times on its own (Table 3).

## Discussion

South Africa has a history of disparity in access to technology and education, presenting challenges in adopting the use of artificial intelligence in the health sector. Digital transformation is growing at a slow rate in the private health sector, and more so in public sector, compared to other industries such as banking and insurance (Willie 2020). This review reflects the slow uptake of machine learning, similar to a review on AI use in the health sector in Tanzania (Sukums et al. 2023), but does show some increasing emergence of machine learning to predict different health outcomes in South Africa. The high number of studies on HIV and/or TB is possibly linked to HIV and/or TB being one of the key burden of diseases (De Villiers 2021; Louw et al. 2023) and those studies reported issues of health-seeking behaviour and compliance with treatment regimens (Stangl et al. 2019). Similarly, the recent COVID-19 pandemic, with its widespread impact, the national requirement of reporting COVID-19 cases for surveillance, and the need for rigorous clinical and societal responses (Van Der Schaar et al. 2021), is also reflected in the studies examined in this review.

There has been numerous reports on the importance of integrating machine learning techniques into local and national healthcare systems to improve health response and health outcomes (Van Der Schaar et al. 2021). However, access to big data routinely collected in South Africa is limited (Tokac et al. 2025a, 2025b), and the review demonstrates the challenges of integrating machine learning into data in the health sector. Firstly, similar to another scoping report (Sukums et al. 2023), the high usage of primary data highlights the challenges of limited access to or availability of large volumes of high-quality data for training and validating AI-based models. Having the availability of the national registries of the NICD and NHLS enables researchers to access these large data sets to apply deep learning to classify images and results and apply conventional and ensemble machine learning.

Secondly, there is lack of data analysis skills in the health sector (Ngiam & Khor 2019). This is similar to reports of challenges related to human, infrastructure, and financial resources for the design, development, and implementation of AI-based solutions in the health sector as reported in Tanzania (Sukums et al. 2023). This is also crucial, considering ethical and medico-legal implications, health workers’ understanding of machine learning tools, and data privacy and security (Ngiam & Khor 2019).

### Implications and recommendations

Overcoming challenges in South Africa requires access to high quality big data, inclusive policies that promote widespread access to the benefits of using AI in healthcare, and AI literacy in the health sector to understand and address ethical and medico-legal implications.

### Strengths and limitations

A strength of this scoping review is its focus on machine learning; however, a limitation is that there is a possibility that important articles might have been missed because of a wide range of machine learning techniques.

## Conclusion

In South Africa, there has been an increase in the use of machine learning in the analysis of health data, but access to big data appears to be a challenge, and disparities have had an impact on the adoption of AI technologies in the healthcare sector.

## Appendices

### Appendix 1

**TABLE 1-A1:** Search terms used to retrieve sources.

PubMed	(((((South Africa[MeSH Terms]) OR (South Africa[Title/Abstract]) OR (South Africa)[Text Word])) AND ((((((Health) OR (Health symptoms[MeSH Terms])) OR (diseases diagnosis[Title/Abstract])) OR (health sector[Title/Abstract])) OR (Health care sector[Title/Abstract])) OR (Disease[Text Word]))) AND (((((((((((((Artificial intelligence [MeSH Terms]) OR (AI[Title/Abstract])) OR (Artificial intelligence[Title/Abstract])) OR (Machine Intelligence[Title/Abstract])) OR (Machine Intelligence)) OR (Computer Reasoning[Title/Abstract])) OR (Deep learning[Title/Abstract])) OR (Machine learning[Title/Abstract])) OR (Artificial intelligence based assessment[Title/Abstract])) OR (Artificial intelligence based assessment[Title/Abstract])) OR (Artificial intelligence-based assessment[Title/Abstract])) OR (Artificial intelligence-based innovations[Title/Abstract])) OR (Artificial intelligence based innovations [Title/Abstract]))
Academic Search Complete	((‘health’/exp OR ‘health’) OR ‘health symptoms’ OR ‘diseases diagnosis’ OR ‘health sector’ OR ‘health care sector’) AND ((‘artificial intelligence’/exp OR ‘artificial intelligence’) OR ‘machine intelligence’ OR (‘automated reasoning’/exp OR ‘automated reasoning’) OR (‘deep learning’/exp OR ‘deep learning’) OR ‘machine learning’ OR ‘artificial intelligence based assessment’ OR ‘artificial intelligence-based innovations’) AND (South Africa)
Google Scholar	(‘health symptoms’ OR ‘diseases diagnosis’ OR ‘health sector’ OR ‘health care sector’ AND ‘artificial intelligence’ OR ‘machine intelligence’ OR ‘automated reasoning’ OR ‘automated reasoning’ OR ‘deep learning’) AND (South Africa)

### Appendix 2

**TABLE 1-A2:** Detailed categorisation of machine learning methods by type.

ML methods	Explanation of acronyms	ML types
SVM, NB, GLM, C5.0, ETC, RF, ANN	SVM (Support Vector Machine), NB (Naive Bayes), GLM (Generalised Linear Model), C5.0 (Decision Tree Model): Conventional ML. ETC: Extra Trees Classifier- RF: Ensemble ML (decision tree-based). ANN: Deep ML (neural networks).	Conventional ML (SVM, NB, GLM, C5.0), Ensemble ML (ETC, RF), Deep ML (ANN).
GPR	Gaussian Process Regression: A probabilistic regression approach.	Conventional ML
GAN	Generative Adversarial Network: Deep learning for synthetic data generation.	Deep ML
XGBoost, Light GBM, GBC, LSTM, RNN	XGBoost (Extreme Gradient Boosting), Ligh GBM (Ligh Gradient Boosting Machine), GBC (Gradient Boosting Classifier): Ensemble ML. LSTM, RNN (Long Short-Term Memory, Recurrent Neural Network): Deep ML.	Ensemble ML (XGBoost), Deep ML (LSTM, RNN).
KNN, SGD, DT, AdaBoost	KNN (K-Nearest Neighbours), SGD (Stochastic Gradient Descent), DT (Decision Tree): Conventional ML. AdaBoost: Ensemble ML.	Conventional ML (KNN, SGD, DT), Ensemble ML (AdaBoost).
CatBoost	Gradient boosting optimised for categorical features.	Ensemble ML
CNN	Convolutional Neural Network: Designed for image processing.	Deep ML
CART, MARS	CART (Classification and Regression Trees): Conventional ML. MARS (Multivariate Adaptive Regression Splines): Ensemble ML.	Conventional ML (CART), Ensemble ML (MARS).
NLP	Natural Language Processing: Algorithms for analysing and processing language data.	Conventional ML

ML, machine learning.
